# Global Leadership Initiative on Malnutrition as a predictor of mortality and prolonged hospitalization in emergency department patients: A prospective cohort study

**DOI:** 10.1002/ncp.70029

**Published:** 2025-09-07

**Authors:** Renata Wolf, Larissa Farinha Maffini, Johnny Galhano dos Santos, Camila Saueressig, Valesca Dall'Alba

**Affiliations:** ^1^ Nutrition Department, Faculty of Medicine Federal University of Rio Grande do Sul Porto Alegre Brazil; ^2^ Graduate Program in Food, Nutrition, and Health Federal University of Rio Grande do Sul Porto Alegre Brazil; ^3^ Graduate Program in Medical Sciences: Endocrinology Federal University of Rio Grande do Sul Porto Alegre Brazil; ^4^ Graduate Program Sciences in Gastroenterology and Hepatology Federal University of Rio Grande do Sul Porto Alegre Brazil; ^5^ Nutrition and Dietetics Division Hospital de Clínicas de Porto Alegre Porto Alegre Brazil

**Keywords:** anthropometry measurements, emergency department, GLIM criteria, hospital malnutrition, subjective global assessment

## Abstract

**Background:**

Early diagnosis of malnutrition is essential for rapid decision‐making regarding nutrition care to improve patient outcomes. We aimed to evaluate the prevalence of malnutrition using the Global Leadership Initiative on Malnutrition (GLIM) criteria and to assess the association of GLIM with 1‐year mortality and length of hospital stay (LOS) in patients admitted to an emergency department (ED).

**Methods:**

Prospective cohort study conducted in the ED of a university hospital. Nutrition assessment included anthropometry, Subjective Global Assessment (SGA), and GLIM criteria. The receiver operating characteristic curves and logistic regression were used for analysis.

**Results:**

The SGA identified 53.9% of patients as malnourished and the GLIM criteria, 50.9%. GLIM showed good accuracy compared with the SGA, with an area under the curve (86.8%) and high sensitivity (84.8%) and specificity (88.8%) values. Malnutrition assessed by SGA demonstrated predictive validity for LOS (odds ratio [OR] = 2.2, 95% CI: 1.4–3.2; *P* < 0.001) and 1‐year mortality (OR = 8.7; 95% CI: 4.3–17.7; *P* < 0.001). Similarly, malnutrition identified by GLIM increased the risk of LOS (OR = 2.2, 95% CI: 1.3–3.9; *P* = 0.007) and 1‐year mortality (OR = 6.7, 95% CI: 3.6–12.5; *P* < 0.001).

**Conclusion:**

GLIM criteria and SGA demonstrated predictive power for LOS and 1‐year mortality. However, the GLIM criteria stand out for adopting more objective and standardized criteria, making it a viable and reproducible approach for diagnosing malnutrition in ED patients.

## INTRODUCTION

Emergency Departments (EDs) vary in formats and capacities and serve patients with different levels of complexity.^1^ Overcrowding is a common issue in ED and can be defined as a situation in which the number of patients waiting for a consultation/diagnosis/treatment exceeds the available resources in this department.^2^ Some studies suggest that overcrowding occurs when >90% of the beds available for treatment are occupied.^2^

Considering this scenario, an American study showed an extremely low prevalence of malnutrition diagnosis in EDs in the United States: 0.7% in 2006, slightly rising to 1.15% in 2014. The prevalence of malnutrition diagnosis was only considered when described in medical records through the International Classification of Diseases.^3^ In contrast, a recently published study, which aimed to assess the feasibility and predictive capacity of five different malnutrition risk screening tools in an ED, observed that the prevalence of malnutrition risk varied between 35% and 49%, depending on the tool used. In the same study, patients with malnutritional risk had a greater risk of prolonged hospitalization and death within 1 year.^4^

Hospital malnutrition can occur because of low food intake, impaired absorption due to illness, or increased metabolic demand.^5^ It is a major public health issue in both developed and emerging countries, with prevalence rates ranging from 40% to 60%, depending on the differences in the populations studied, assessment methods, and characteristics of each institution.^5^ Also, hospital malnutrition negatively impacts a patient's prognosis by increasing morbidity, mortality, length of hospital stay (LOS), and treatment costs.^6^ Although there are different validated instruments to assess and identify malnutrition, there is no globally recommended instrument for diagnosing malnutrition that can accurately describe its prevalence.^7^

With the aim of unifying criteria and building a global consensus, the Global Leadership Initiative on Malnutrition (GLIM) group developed a proposal for diagnosing malnutrition.^8^ The GLIM consists of three phenotypic criteria (unintentional weight loss, low body mass index [BMI], and reduced muscle mass) and two etiological criteria (reduced food intake or assimilation and inflammation or disease burden). For the diagnosis of malnutrition, at least one phenotypic and one etiological criterion must be met.^8^ Since their publication, the GLIM criteria have been widely studied across various patient populations.^9^, ^10^, ^11^ However, few studies have evaluated its use specifically in EDs.

Early diagnosis of malnutrition is essential for rapid decision‐making regarding nutrition care and for improving patient outcomes. Therefore, the objective of the present study was to assess the prevalence of malnutrition using the GLIM criteria and to evaluate its association with 1‐year mortality and LOS in patients admitted to the ED.

## METHODS

This is a prospective cohort study at the ED of a university hospital, approved by the Hospital de Clinicas de Porto Alegre Research Ethics Committee. All participants included in the study or their caregiver/family member signed the informed consent form before data collection.

The study included both male and female patients admitted to the ED within the previous 24–72 h and aged ≥19 years. Pregnant women, individuals who had undergone limb amputation, comatose or unconscious patients, with no possibility of communication (intubated and sedated) and with mental confusion, were not included.

It is important to emphasize that the ED where data collection was conducted is a reference location for the care of patients with chronic diseases (diabetes, hypertension, heart disease, nephropathy, liver disease, and cancer); trauma cases and burn injuries are not directed to this location.

### Data collection

Two registered dietitians (RDs) with extensive experience in nutrition assessment and two undergraduate students supervised and previously trained to ensure standardization were responsible for malnutrition risk screening, conducting the Subjective Global Assessment (SGA), and performing anthropometric measurements.

Patient selection was performed through daily consultation of the list of admissions to the ED. From this list, patients who met the inclusion criteria were invited to participate in the study. Assessments were conducted at the bedside, preferably in the presence of a family member or caregiver.

**Figure 1:**
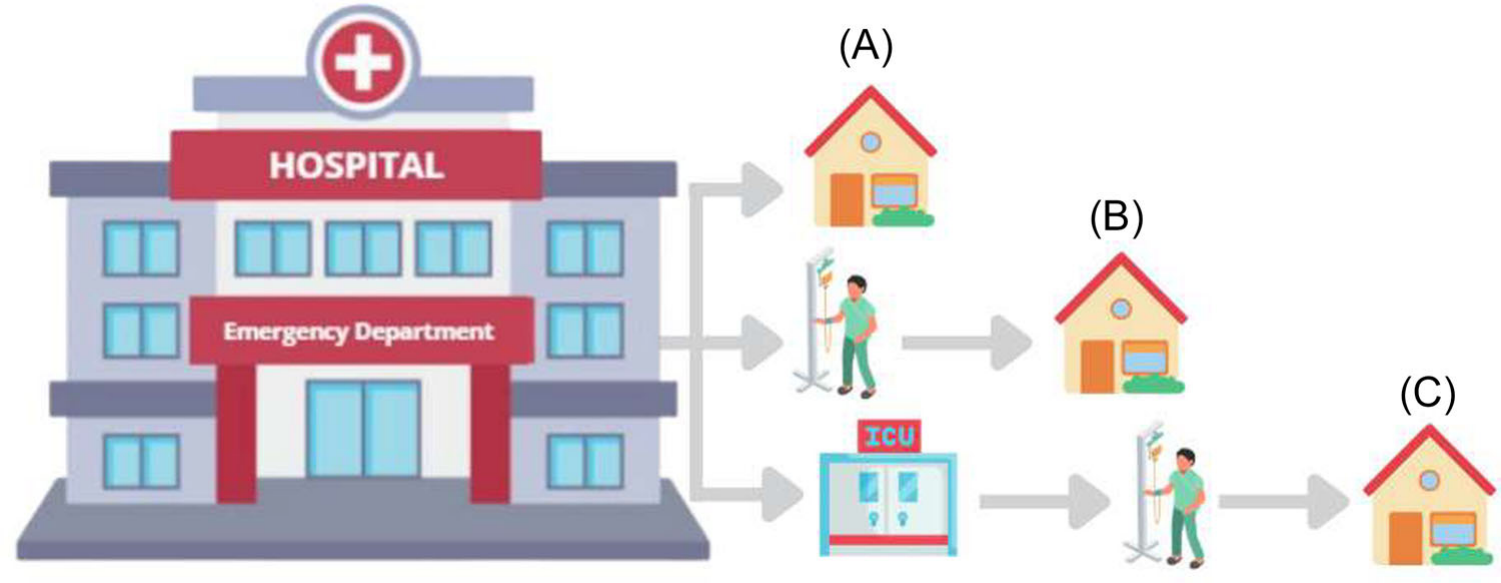
Patient flow from admission through the emergency department to hospital discharge. Overview of patient flow from hospital admission to discharge. Flow A: Admission via the emergency department and direct discharge home. Flow B: Admission via the emergency department transfer to a ward bed, and discharge home. Flow C: Admission via the emergency department, transfer to an intensive care unit bed, subsequent transfer to a ward bed, and discharge home.

As shown in Figure 1, regarding patient discharge, some were discharged directly from the ED to their homes, whereas others required hospitalization in a clinical ward or intensive care unit (ICU) before being discharged. After discharge, all patients were monitored for clinical outcomes through telephone calls, using a predefined form developed by the researchers,and through review of electronic medical records.

### Demographic and clinical data

Demographic and clinical data (date of birth, age, sex, ethnicity, reason for hospitalization, and previous pathologies) were collected from patients' electronic medical records. Preexisting comorbidities were assessed using the Charlson Comorbidity Index. The Charlson Comorbidity Index consists of a list of 16 predefined comorbidities, which have specific scores. The patient's age should be considered when >40 years. For the calculation of the index, the scores related to the comorbidities presented by the patient should be summed, and the age, when applicable, should be added to the total.^12^

### Anthropometric assessment

All patients were weighed by the researchers using a portable scale at the bedside to determine their current weight. Patients with ascites or edema had their weight corrected, according to the degree of edema and/or ascites.^13^ For patients confined to bed, it was necessary to estimate their height and weight. Height was estimated using the formula proposed by Chumlea,^14^ which uses the knee height measurement. To estimate weight, arm circumference and knee height were measured for subsequent calculation using a mathematical formula also proposed by Chumlea.^15^

BMI was considered low when <18.5 for adults^16^ and <22 for older adults.^17^ Patients reported their usual weight. To calculate the percentage of weight loss, the formula of Blackburn et al^18^ was used: [(usual weight ‐ current weight/usual weight) x 100].

Mid‐upper arm circumference (MUAC) was measured at the midpoint between the acromion and the olecranon, with a nonstretchable millimeter tape, and assessed using the values proposed by Frisancho^19^ as reference. Calf circumference (CC) was measured at the point of greatest circumference, using a nonstretchable millimeter tape. After measurement, CC was adjusted according to BMI: 3 cm of the measured CC were reduced for BMI from 25 to 29.9, 7 cm for BMI from 30 to 39.9, and 12 cm for BMI of ≥40.^20^ For the CC to be considered as reduced muscle mass, the following cutoff points were used: ≤33 cm for women and ≤34 cm for men.^21^

### Malnutrition risk screening

Three tools to identify malnutrition risk were used in the study: Malnutrition Universal Screening Tool (MUST),^22^ Nutritional Risk Emergency 2017 (NRE‐2017)^23^ and Nutritional Risk Screening 2002 (NRS‐2002).^24^

MUST focuses on identifying the risk of malnutrition in hospitalized patients through BMI, weight loss in 3–6 months, and reduced food intake; the final score classifies the nutrition risk as low (0 points), medium (1 point), or high (≥2 points).^22^

NRE‐2017 was developed by Brazilian researchers in an ED. It takes into account age >65 years, reduced food intake, food consistency, weight loss, and muscle loss identified on physical examination. The score ranges from 0 to 2.5, with scores of ≥1.5 being considered at risk.^23^

NRS‐2002 considers factors such as BMI, weight loss, reduced food intake, and severity of the disease. An additional point is added for patients aged ≥70 years. A score of ≥3 points classifies the patient as being at risk.^24^

### Malnutrition diagnosis

All the patients included in the study underwent a full nutrition assessment. The Subjective Global Assessment (SGA), consisting of questions involving weight loss, reduced food intake, presence of gastrointestinal symptoms, functional capacity, and physical examination, was the gold standard tool used. It classifies patients as well‐nourished (A), moderately (or suspected of being) malnourished (B), or severely malnourished (C).^25^

**Figure 2:**
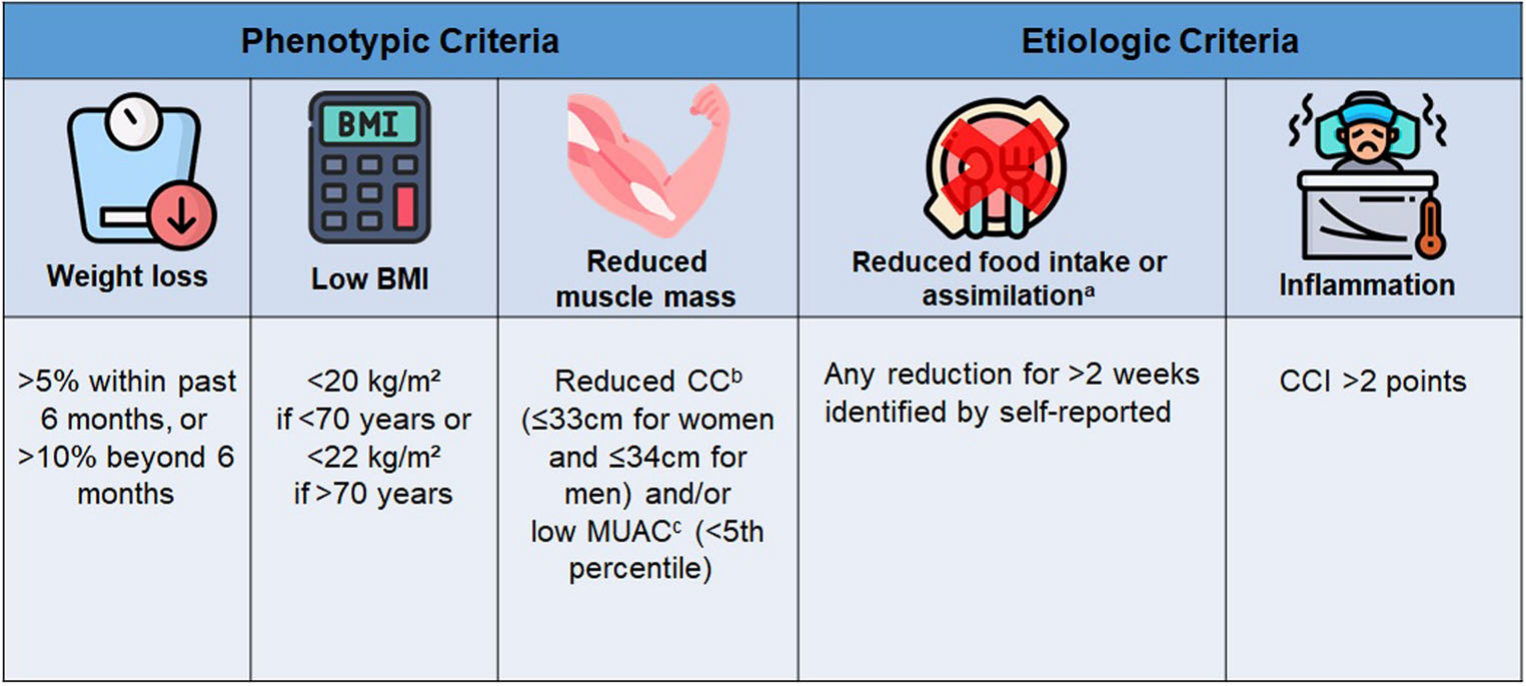
Cutoff points used to diagnose malnutrition in emergency patients according to GLIM criteria. ^a^Any reduction for >2 weeks identified by self‐reported percentage of actual food intake (100%, 75%, 50%, 25%, or 0%) compared with their usual intake or gastrointestinal symptoms (ie, nausea, vomiting, and diarrhea) or any chronic gastrointestinal condition that adversely impacts food intake/absorption/assimilation (ie, pancreatitis, inflammatory bowel disease) registered in electronic records. ^b^Reduced CC: ≤34 cm for men and ≤33 cm for women.^21^ CC values were adjusted for BMI to help remove the confounding effects of adiposity.^20^ ^c^Low MUAC: <5th percentile.^26^ CC, calf circumference; CCI, Charlson Comorbidity Index; GLIM, Global Leadership Initiative on Malnutrition; MAC, mid‐arm circumference.

The GLIM criteria were also applied, and for the diagnosis of malnutrition at least one phenotypic criterion and one etiological criterion had to be met, as shown in Figure 2.

The aim of this study was to compare malnutrition diagnosed both by the SGA and by the GLIM criteria. Patients classified as moderately malnourished (B) and severely malnourished (C) by the SGA and GLIM were grouped into a single category (malnourished).

### Sample size calculation

**Figure 3:**
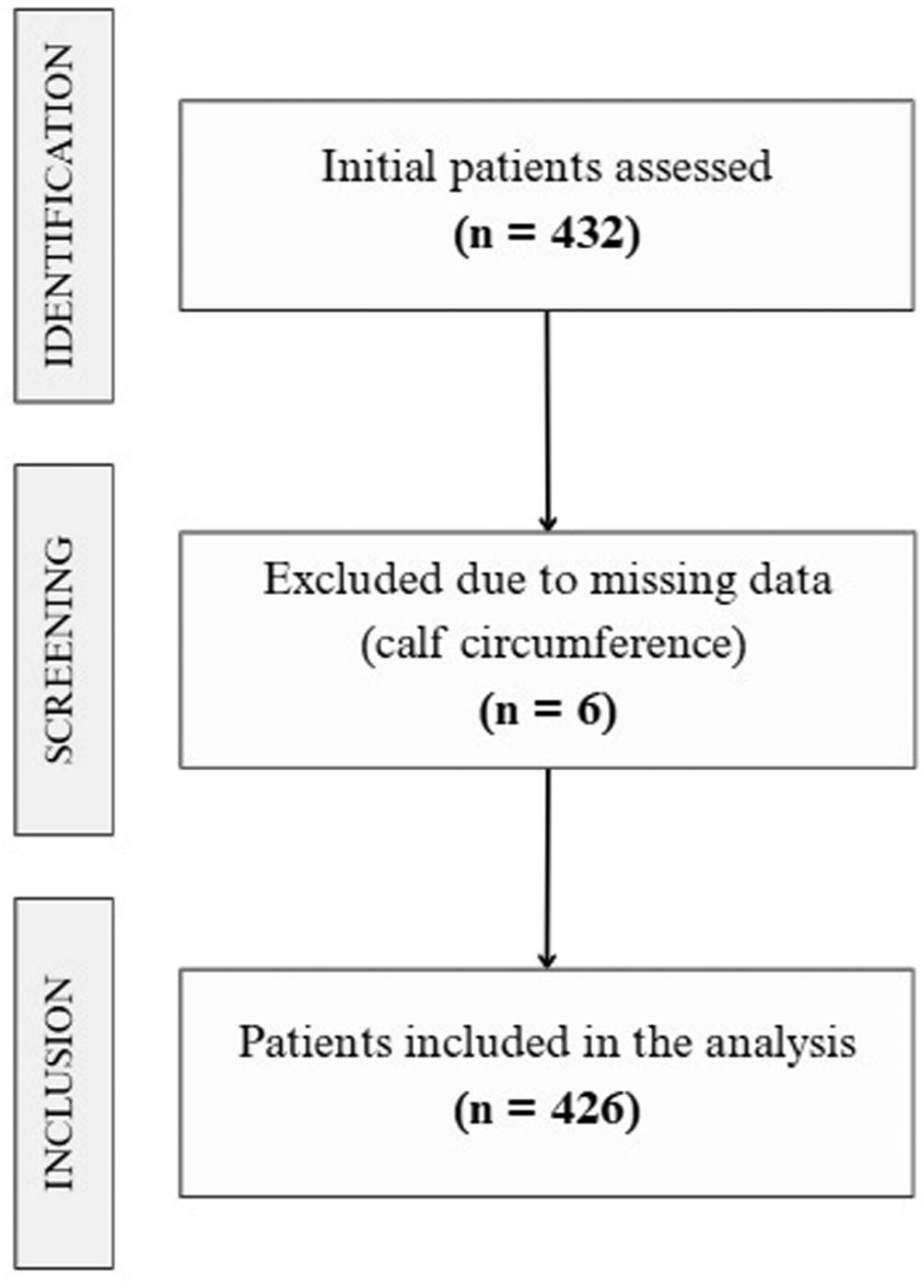
Flowchart of the patient selection process.

For the original study, which was designed to evaluate different screening tools for malnutrition risk in the ED, a sample size of 432 was calculated.^4^ During the collection period (March to October 2019), patients were included consecutively. For the analyses of this study, 426 of these patients were selected, as shown in Figure 3.

The post hoc statistical power to detect an odds ratio (OR) of 6.7 in the association between malnutrition and mortality, with a significance level of 5%, was 100%, considering 217 individuals in the malnourished group, 209 in the well‐nourished group, and a mortality prevalence of 6.7% in the well‐nourished group. The calculation was performed using the Epi Info software.

### Statistical analyses

Continuous variables were presented as mean and SD or median and interquartile range (IQR) according to distribution, and categorical variables were presented as their absolute and relative frequencies. Normality of the variables was assessed using the Kolmogorov‐Smirnov test. Comparisons between patients with and without malnutrition according to the GLIM were made using the Student *t* test or Mann‐Whitney and chi‐square tests.

The agreement between the diagnosis of malnutrition using the GLIM criteria and the SGA was analyzed using the kappa coefficient (k). The values were classified as follows: 0.20 as poor, 0.21–0.40 as fair, 0.41–0.60 as moderate, 0.61–0.80 as substantial, 0.81–0.99 as almost perfect, and 1.00 as perfect.^27^ The area under the receiver operating characteristic (ROC) curve with a 95% CI and the 95% sensitivity, specificity, and positive and negative predictive values were determined to investigate the concurrent validity of the GLIM criteria using SGA as the reference method. Sensitivity and specificity values of >80% were required to consider the concurrent validity satisfactory.^8^

Multiple logistic regression analysis was used to calculate the OR and respective 95% CI to test the predictive validity of malnutrition diagnosed by the GLIM criteria. The dependent variables included LOS (≥6 days, corresponding to the median length of stay in the total sample, including patients admitted only to the ED as well as those transferred to the ward or ICU), 1‐year mortality, and transfer to ICU. All models were adjusted for sex and age. The significance level adopted was 5%. Statistical analyses were performed in the SPSS, version 18, statistical package.

## RESULTS

Table 1 describes the characteristics of the 426 patients included in this study (mean age 57.3 ± 15.6 years, 54% female, and 82.9% White). The GLIM criteria identified 50.9% (*n* = 217) of the patients as malnourished, whereas the SGA identified a prevalence of 53.9% (*n* = 230).

**Table 1:** Malnutrition diagnosis according to the GLIM criteria and SGA.

		GLIM		SGA	
Variables	Whole sample (*n* = 426)	Well‐nourished (*n* = 209)	Malnourished (*n* = 217)	Well‐nourished (*n* = 196)	Malnourished (*n* = 230)
**General features**					
Age (years)	57.3 ± 15.6	53 ± 15	61 ± 15	54 ± 16	60 ± 15
Sex (female)	230 (54%)	106 (50.7%)	124 (57.1%)	97 (49.5%)	133 (57.8%)
Ethnicity (White)	353 (82.9%)	171 (81.8%)	182 (83.9%)	163 (83.2%)	190 (82.6%)
**Clinical features**					
Comorbidities (yes)^a^	381 (89.4%)	179 (85.6%)	202 (93.2%)*	161 (82.1%)	220 (95.7%)*
**Clinical outcomes**					
LOS (days)	6 (3–11)	4 (3–9)	7 (3–12)	4 (3–9)	7 (3–12)
LOS ≥ 6 days	220 (51.6%)	30 (43.5%)	130 (59.9%)*	83 (42.3%)	137 (59.6%)*
Death within 1 year	91 (21.4%)	14 (6.7%)	77 (35.5%)*	10 (5.1%)	81 (35.2%)*
ICU admission	23 (5.9%)	10 (4.8%)	13 (6%)	8 (4.1%)	15 (6.5%)
**Nutrition features**					
Current weight (kg)	70.8 ± 18.7	77.3 ± 18.9	64.8 ± 16.2*	77.8 ± 19.8	65 ± 15.4*
BMI (kg/m^2^)	26 ± 6.3	28.2 ± 6.5	23.9 ± 5.3*	28.5 ± 6.6	24 ± 5.29*
Low BMI (kg/m^2^)^b^	70 (16.4%)	16 (7.7%)	54 (24.9%)*	13 (6.6%)	57 (24.8%)*
NRS‐2002 ≥ 3	156 (36.6%)	20 (10.2%)	136 (59.1%)*	13 (6.6%)	57 (24.8%)*
MUST ≥ 2	200 (46.9%)	40 (19,1%)	160 (73.7%)*	29 (14.8%)	171 (74.3%)*
NRE‐2017 ≥ 0.75	191 (44.8%)	31 (14.8%)	160 (73.7%)*	22 (11.2%)	169 (73.5%)*

*Note*: Data are presented as number (%), media±SD or median (P25‐P75). BMI is calculated as weight in kilograms divided by height in meters squared.

*P* value by the chi‐square test, Fisher exact test, Student *t* test, and Mann‐Whitney test.

Abbreviations: BMI, body mass index; ICU, intensive care unit; LOS, length of stay; MUST; Malnutrition Universal Screening Tool; NRE‐2017, Nutritional Risk Emergency 2017; NRS‐2002, Nutritional Risk Screening 2002.

* *P* value < 0.05: comparison between the groups (well‐nourished and malnourished) according to GLIM and SGA.

^a^ Comorbidities: diabetes, cardiovascular disease, kidney disease, liver disease, cancer, human immunodeficiency virus and hypertension.

^b^ Low BMI: <18.5 for adults^16^ and <22 for older adults.^17^

At least one chronic condition (such as diabetes, cardiovascular disease, kidney disease, liver disease, cancer, or hypertension) was present in 89.4% (*n* = 381) of this sample. Patients identified as malnourished by SGA and GLIM had a higher prevalence of comorbidities when compared with well‐nourished patients (*P* < 0.05).

Mean weight at the time of hospital admission was 70.8 ± 18.7 kg, whereas BMI was 26 ± 6.3. Low BMI was present in 16.4% (*n* = 70) of the sample. Those identified as malnourished by SGA and GLIM on admission had lower mean weights, lower BMI, and a higher frequency of low BMI when compared with well‐nourished patients (*P* < 0.05).

The NRS‐2002 tool identified a prevalence of malnutrition risk at 36.6% (*n* = 156), MUST at 46.9% (*n* = 200), and NRE‐2017 at 44.8% (*n* = 191). Patients considered malnourished by both the SGA and GLIM had a higher prevalence of malnutritional risk for all the tools when compared with the group of well‐nourished patients (*P* < 0.05).

The median LOS was 6 days (IQR: 3–11), and 51.6% (*n* = 220) of the patients had a prolonged hospital stay. Among patients admitted to the ED, 5.9% (*n* = 23) experienced clinical deterioration during their hospital stay and required ICU admission. Death within 1 year occurred in 21.4% (*n* = 91) of cases. Malnourished patients according to the SGA and GLIM showed a longer hospital stay and a higher prevalence of death when compared with the group of well‐nourished patients (data shown in Table 1).

Table 2 shows the agreement and accuracy of the GLIM criteria for identifying malnutrition, using the SGA as a reference. The GLIM criteria presented good accuracy with AUC (86.8%), with high sensitivity (84.8%) and specificity (88.8%) values. The agreement between the GLIM criteria and the SGA was considered substantial with a kappa value of 0.732 (*P* < 0.001).

**Table 2:** Accuracy and agreement of the GLIM criteria to predict malnutrition in an emergency department, using SGA as the reference standard.

	Malnutrition^a^		Agreement^c^ (Kappa)	Validity^d^	
	AUC ROC^b^ (95% CI)	Accuracy (%)		Sensitivity (%)	Specificity (%)
**GLIM criteria**	0.868 (0.831–0.905)	86.8	0.732	84.8	88.8

Abbreviations: AUC, area under the curve; GLIM, Global Leadership Initiative on Malnutrition; ROC, receiver operating characteristic.

^a^ Prevalence of malnutrition according to Subjective Global Assessment (reference standard) = 54%.

^b^ AUC values = 0.5–0.6 (very poor), 0.6–0.7 (poor), 0.7–0.8 (moderate), 0.8–0.9 (good), >0.9 (excellent).

^c^ Kappa values = 0.20 (poor), 0.21–0.40 (fair), 0.41–0.60 (moderate), 0.61–0.80 (substantial), 0.81–0.99 (almost perfect), 1.00 (perfect).

^d^ Sensitivity, specificity, positive predictive value, and negative predictive value cutoffs: 90% to 100% (high), >80% to ≤89% (moderate); ≤79% (low).

As shown in Table 3, patients classified as malnourished according to the SGA showed significantly worse clinical outcomes. Malnutrition identified by SGA was associated with a higher likelihood of LOS (OR = 2.2, 95% CI: 1.4–3.2; *P* < 0.001). In addition, SGA demonstrated strong predictive value for 1‐year mortality (OR = 8.7, 95% CI: 4.3–17.7; *P* < 0.001). However, no statistically significant association was found between SGA‐defined malnutrition and clinical deterioration requiring ICU admission (OR = 1.3, 95% CI: 0.5–3.6; *P* = 0.541). Patients diagnosed with malnutrition according to GLIM criteria also showed worse clinical outcomes. Malnutrition identified by the GLIM criteria increased the risk of LOS (OR = 2.2, 95% CI: 1.3–3.9; *P* = 0.007) and 1‐year mortality (OR = 6.7, 95% CI: 3.6–12.5; *P* < 0.001). No statistically significant association was found between GLIM criteria–defined malnutrition and clinical deterioration requiring ICU admission (OR = 1.2, 95% CI: 0.5–2.8; *P* = 0.009). In both analyses, the models were adjusted for confounding factors such as sex and age.

**Table 3:** Predictive validity of SGA and the GLIM criteria for malnutrition diagnosis: logistic regression analysis.

Dependent variable	OR (95% CI)*	*P* value
**SGA**		
**LOS (**≥**6 days)**		
Crude	2.0 (1.4–3.0)	<0.001
Adjusted*	2.2 (1.4–3.2)	<0.001
**Death within 1 year**		
Crude	9.9 (4.9–19.8)	<0.001
Adjusted*	8.7 (4.3–17.7)	<0.001
**ICU admission**		
Crude	1.5 (0.6–3.8)	0.415
Adjusted*	1.3 (0.5–3.6)	0.541
**GLIM**		
**LOS (**≥**6 days)**		
Crude	1.9 (1.1–3.4)	0.02
Adjusted*	2.2 (1.3–3.9)	0.007
**Death within 1 year**		
Crude	7.8 (4.3–14.4)	<0.001
Adjusted*	6.7 (3.6–12.5)	<0.001
**ICU admission**		
Crude	1.3 (0.5–2.9)	0.001
Adjusted*	1.2 (0.5–2.8)	0.009

Abbreviations: GLIM, Global Leadership Initiative on Malnutrition; ICU, intensive care unit; LOS, hospital length of stay; OR, Odds Ratio; SGA, Subjective Global Assessment.

* Model adjusted for sex and age.

## DISCUSSION

In the present study, the GLIM criteria showed good agreement and high accuracy (almost 90%), sensitivity and specificity values (>80%) when compared to the SGA. Furthermore, patients diagnosed as malnourished by the GLIM increased the risk of LOS by approximately 2.2 times and 1‐year mortality by 6.7 times.

There is considerable variability in the prevalence of malnutrition in hospitalized patients, with studies reporting rates ranging from 16.7% to 80% using the GLIM.^28^, ^29^, ^30^, ^31^, ^32^ Studies focused on emergency admissions show narrower variations, ranging from 50.3%^7^ to 53.7%,^33^ which is consistent with the findings of the present study.

In this study, the GLIM phenotypic criteria used were percentage of weight loss, BMI, MUAC, and CC. Two other groups of researchers conducted similar studies but adopted different parameters. Speranza et al used percentage of weight loss and low BMI as phenotypic criteria but also included fat‐free mass index (calculated as weight in kilograms divided by height in meters squared) and the presence of acute inflammation related to disease or injury in their assessment.^33^ According to the GLIM guideline,^34^ although the inflammation criterion is valid, it is an etiological, not a phenotypic, criterion. On the other hand, Fernandez et al evaluated a sample composed exclusively of critically ill older adult patients in the ED and applied anthropometric measurements such as CC, MUAC, percentage of weight loss, and low BMI as phenotypic criteria.^7^

Although anthropometric measurements are not the reference method in the GLIM phenotypic criteria evaluation guide, because of the presence of edema and obesity, which would be classified as a confounding effect, in this study, the new cutoff points for CC were used to correct the overweight confounding effect,^21^ and patients with anasarca were excluded. It is well‐known that in an environment with high patient turnover and challenges in implementing fasting protocols, anthropometric measurements offer an easy, quick, and low‐cost method for evaluating muscle mass when compared with imaging tests and the application of bioimpedance analysis.^34^

To date, we have identified in the literature only one study evaluating the concurrent and predictive validity of the GLIM criteria in patients admitted to an ED. The study by Fernandez et al used the Mini Nutritional Asessment–Short Form as a reference, given that the sample consisted exclusively of older adult patients and showed good accuracy, with moderate sensitivity and specificity, but still lower values than our findings. The authors also evaluated different outcomes, demonstrating that malnutrition, diagnosed by the GLIM criteria, increased the risk of transfer to the ICU and that the presence of severe malnutrition was associated with in‐hospital mortality.^7^

Other studies conducted with hospitalized patients in different specialties have also shown an increased chance of negative outcomes in malnourished patients according to the GLIM criteria using the SGA as a reference.^35^, ^36^

In recent years, several studies have reinforced the validity of the GLIM criteria in identifying malnutrition.^10^, ^11^, ^35^ Although the SGA is one of the most recognized and widely used tools for evaluation in various contexts, it has some limitations, especially due to its subjectivity and the need for highly trained evaluators. Another important aspect is that SGA is not very sensitive for short‐term monitoring and does not include objective assessment of muscle mass, a significant limitation given that many patients may present with malnutrition associated with obesity or overweight. On the other hand, the GLIM criteria include a specific phenotypic variable for the assessment of muscle mass, representing an advance over subjective tools.^37^ The GLIM consensus itself recognizes that adults with obesity may require specific adjustments in the assessment of muscle mass, since excess adiposity can confound the interpretation of results.

Some constraints inherent to this study must be considered, such as missing data due to restrictions in the nutrition assessment of bedridden or unaccompanied patients, as well as the difficulty in obtaining accurate self‐reported data. In addition, we lack data regarding nutrition therapy during hospitalization. However, it is important to highlight that the primary objective of this study was to verify to what extent the nutrition status of the patient on arrival at the hospital—that is, prior to dietary interventions—could impact mortality.

On the other hand, the inclusion of a heterogeneous sample, consisting of a wide variety of diagnoses, represents the complexity of the patients who access the ED and reflects the reality of the situations commonly encountered in routine care.

These findings reinforce the clinical utility of the GLIM in the ED and support its adoption as a valid tool for assessing nutrition status in these patients. This study makes a relevant contribution to the literature by systematically evaluating the diagnostic and predictive validity of the GLIM instrument in hospital EDs. Although previous studies have explored the topic in this context, we have observed the methodological limitations previously mentioned. This study includes a broad population (adult and older adult patients) and presents novel outcomes not yet explored in previous studies with emergency patients. The application of an appropriate method (sensitive and specific) for diagnosing malnutrition can assist professionals in clinical practice, contributing to more appropriate and individualized nutrition care.

## CONCLUSION

In conclusion, the GLIM criteria showed good diagnostic and predictive validity. The diagnosis of malnutrition was associated with an increased risk of LOS and 1‐year mortality. In addition, GLIM showed almost perfect agreement and good sensitivity and specificity when compared to the SGA. Therefore, the GLIM criteria can be used to diagnose malnutrition in the ED.

## AUTHOR CONTRIBUTIONS

Valesca Dall'Alba contributed to the conception and design of the research. Renata Wolf, Larissa Farinha Maffini, Johnny Galhano dos Santos, Camila Saueressig, and Valesca Dall'Alba contributed to the acquisition, analysis and interpretation of the data. All authors drafted the manuscript, critically revised the manuscript, agree to be fully accountable for ensuring the integrity and accuracy of the work, and read and approved the final manuscript.

## CONFLICT OF INTEREST STATEMENT

None declared.

## Supporting information

Supporting information
